# Risk Factors for Contralateral Occult Papillary Thyroid Carcinoma in Patients with Clinical Unilateral Papillary Thyroid Carcinoma: A Case-Control Study

**DOI:** 10.1155/2022/5112985

**Published:** 2022-06-28

**Authors:** Liu Yihao, Li Shuo, Xi Pu, Wang Zipeng, Sun Hanlin, Chang Qungang, Wang Yongfei, Yin Detao

**Affiliations:** ^1^Thyroid Surgery Department, The First Affiliated Hospital of Zhengzhou University, Zhengzhou, China; ^2^Engineering Research Center of Multidisciplinary Diagnosis and Treatment of Thyroid Cancer of Henan Province, Zhengzhou, China; ^3^Key Medicine Laboratory of Thyroid Cancer of Henan Province, Zhengzhou, China

## Abstract

**Introduction:**

Papillary thyroid cancer (PTC) is one of the most prevalent endocrine malignancies that has increased in recent decades around the world. Although the indicator for navigating the surgical extent in PTC patients is still in debate, a key issue is how to predict that there are undetected preoperative tumors in the contralateral thyroid lobe. This study aims to find risk factors for contralateral occult papillary thyroid cancer (COPTC) to facilitate more accurate surgical decisions made for patients with PTC.

**Materials and Methods:**

In our study, we included 229 patients who underwent total thyroidectomy plus central and ipsilateral lateral lymph nodes dissection from January 1, 2019, to September 1, 2021. Univariate and multivariate logistic regression analyses were conducted to assess the association between COPTC and clinical-pathological characteristics, as well as the relation between the diameter of the occult lesions and predictors. The forest plot was plotted to visualize the prediction factors from the output of the multivariate regression analysis. A ROC curve was used to evaluate the combining potency of all the risk factors.

**Results:**

Of the 229 patients included in our study, 46 with COPTC were assigned to the case group, representing 20.1% in this study. Multifocality in one lobe (OR = 2.21, *P*=0.03), intact capsule (OR = 2.54, *P*=0.01), central lymph node metastasis (OR = 3.00, *P*=0.02), and Hashimoto's thyroiditis (OR = 2.08, P = 0.04) are more prone to present contralateral occult papillary thyroid carcinoma. The ROC curve of the aggregate potency of the risk factors presents AUC = 0.701 (*P* < 0.001), and the best cutoff value was 2.02, with a sensitivity of 78.3% and specificity of 55.2%. Furthermore, there was no statistical correlation between the diameter of the occult tumor and the four obtained variables.

**Conclusion:**

Patients with multifocality in one lobe, intact capsule, central lymph node metastasis, and HT may harbor contralateral papillary thyroid carcinoma. It is essential to be prudent to make a surgical or follow-up decision on these patients. In addition, more clinical rather than postoperative pathological indicators need to be revealed in the future.

## 1. Introduction

Thyroid cancer, as one of the most common malignant endocrine neoplasms, has increased in all parts of the world in recent decades [1], which mainly results from the prevalence of differentiated thyroid carcinoma (DTC), which accounts for above 90% of all types of thyroid carcinoma, and papillary thyroid carcinoma(PTC) is the most common pathological subtype of DTC [2,3]. Therefore, many emerging studies on the diagnosis, treatments, prognosis, biomarkers, and genomics of PTC have been helpful in the diagnosis and treatment. Fine-needle aspiration biopsy (FNAB) navigated by Doppler ultrasound combined with genetic tests of BRAFv600e and RAS can help discriminate poorly defined nodules by imaging examinations [4,5]; serum thyroglobulin (sTg) can also contribute to the diagnosis and monitoring of recurrence [6,7], and serum thyroid-stimulating hormone (TSH) can also be a valuable tool for the management and follow-up of patients who underwent surgery [8]. Generally, in fact, the prognosis of PTC is excellent based on precise diagnosis, normative operations, TSH suppression therapy, and postoperative iodine radiotherapy over the years, and the mortality and occurrence remain stable [1], while also partly due to which may be its inertness.

Preoperative Doppler ultrasound plays an essential role in the detection of thyroid nodules for an economical and convenient reason instead of computed tomography (CT), magnetic resonance imaging (MRI), or 18F-FDG-PET scans [9]. By describing the size, location, calcification type, margin, blood supply, and lymph node morphology of the neck region from skilled sonographers, surgeons can preliminarily judge the necessity and method of surgery. However, for those micronodules, usually 5 mm or less, even high-resolution ultrasounds are challenging to detect [10]. Limitations of preoperative ultrasound can omit some occult lesions, regardless of malignant or benign nodules.

Surgery is undoubtedly the irreplaceable way to treat most papillary thyroid cancers and aims to eliminate primary tumors, reduce the risk of cancer recurrence, and allow postoperative iodine therapy, if necessary. However, the choice of lobectomy and total thyroidectomy remains a dilemma. According to the 2015 ATA guidelines, for tumors lesser than 4 cm, confined to one lobe, absent of extrathyroidal extension (ETE), and clinical lymph nodes metastasis (LNM), the extent of surgery was recommended as either lobectomy or total thyroidectomy (TT) [11], depending on the intentionality from patients and surgery team. However, some scholars argued that total thyroidectomy has the advantage of allowing individualized postoperative risk stratification and surveillance [12], and some other previous studies have reported that in patients with unilateral thyroid cancer who underwent total thyroidectomy, contralateral malignant tumors, defined as occult thyroid carcinoma, also represented a nonnegligible proportion [13–17]. Furthermore, evidence indicates that multifocality, including occult contralateral or bilateral thyroid cancer, is an independent risk predictor of recurrence after lobectomy [18]. Indetermination of contralateral occult thyroid cancer risk factors results in unnecessary bilateral thyroid dissection or insufficient surgery. Some researchers have tried to seek contralateral thyroid cancer risk factors, while the evidence obtained is still limited. As a result, we conducted this study to explore the predictors of contralateral occult papillary thyroid carcinoma (COPTC) in clinical unilateral thyroid cancer patients based on the ultrasound and pathological characteristics of the primary detected lesions in a circumstance of time-consuming debating between total and unilateral thyroidectomy.

## 2. Materials and Methods

### 2.1. Definitions

We define unilateral papillary thyroid cancer as malignant nodules located in only one thyroid lobe. Then, contralateral occult papillary thyroid cancers (COPTC) were defined as malignant tumors undetected by preoperative imaging, mainly Doppler ultrasound, located in the contralateral lobe of the primary unilateral carcinoma detected by at least two pathologists postoperatively. The location (upper or other) and diameter of the primary largest tumor foci were based on preoperative Doppler ultrasound examination description, which was reported by at least one experienced ultrasound physician. Furthermore, multifocality of ipsilateral carcinoma, the diameter of the contralateral occult lesions, capsular status, tracheal invasion, strap muscle invasion, central lymph node metastasis (CNM), and lateral lymph node metastasis (LNM) were based on postoperative paraffin section diagnosed by at least two experienced pathologists.

### 2.2. Samples

**Figure 1:**
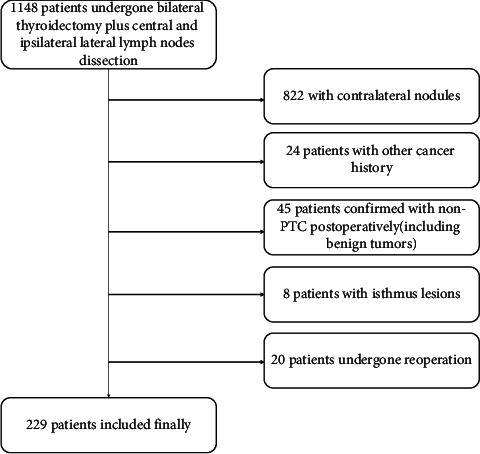
Flowchart illustrating the selection of the samples.

This retrospective case-control study consisted of patients in a high-volume thyroid surgery unit of The First Affiliated Hospital of Zhengzhou University, who underwent total thyroidectomy plus bilateral central and ipsilateral lateral lymph node dissection from January 1, 2019, to September 1, 2021. Patients with the following conditions were excluded: (1) contralateral undetermined nodules detected by preoperative image examination or pathology; (2) distant metastasis at diagnosis on pathological or clinical analysis; (3) history of other malignant tumors; (4) history of head and neck radiation exposure; (5) non-PTC confirmed by postoperative pathology (follicular, medullary, anaplastic, or benign); (6) recurrence for reoperation; and (7) lesions located in the isthmus lobe. Finally, we had 229 patients included in this study, in which 46 patients who were diagnosed with bilateral thyroid papillary cancer postoperatively were brought into the case group, which means they had COPTC, and 183 patients with unilateral PTC with or without contralateral benign nodules were assigned to the control group. This process is shown in Figure 1. Clinical pathological characteristics such as age, sex, body mass index (BMI), ipsilateral multifocality of the primary tumor, location and diameter of the primary largest tumor foci, diameter of the contralateral lesions, capsular status, trachea invasion, strap muscles invasion, central lymph nodes metastasis (CNM), ipsilateral lateral lymph nodes metastasis (LNM), BRAFv600e mutation, serum thyroglobulin (Tg), and presence of Hashimoto's thyroiditis (HT) were collected.

### 2.3. Statistical Analysis

All statistical analyses were performed by SPSS v26.0 software (Chicago, IL, USA). Continuous variables were expressed as means ± standard deviations (SD), and classified variables were presented as frequency and percentages. Student's t-test and Pearson's chi-square test were conducted for the comparison between the case and control groups. Univariate logistic regression analyses were performed to initially identify risk factors for contralateral thyroid cancer. Then, all statistically significant variables in univariate analyses were included in multivariate logistic regression analyses to find the optimal COPTC indicators. The forest plot was plotted by using GraphPad Prism 9 to visualize the prediction factors from the output of the multivariate regression analysis. A ROC curve was used to evaluate the combining potency of all the risk factors. Finally, we also evaluated the association between the diameter of the contralateral lesions and the four variables.

## 3. Results

Table 1 presents the distribution of clinical and pathological characteristics between the case and control groups. Of all patients included in our study, 159 were women and 70 were men, aged 15 to 71 years, and the incidence of COPTC in the patients was 20.1% (46 cases). No significant difference was found in age, gender, BMI, location, diameter, trachea invasion, strap muscle invasion, B-RAF mutation, and serum Tg. Yet there were significant differences with respect to ipsilateral multifocality (52.2% vs 33.3%, *P* < 0.05), intact capsule (43.5% vs 27.7%, *P* < 0.05), central lymph nodes metastasis (84.8% vs 68.9%, *P* < 0.05), and Hashimoto's thyroiditis (43.5% vs 27.3%, *P* < 0.05).

**Figure 2:**
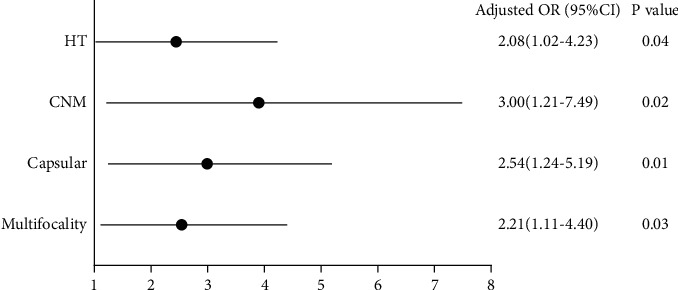
Forest plot of the significant factors in multivariate logistic regression of predicting contralateral occult papillary thyroid cancer.

**Figure 3:**
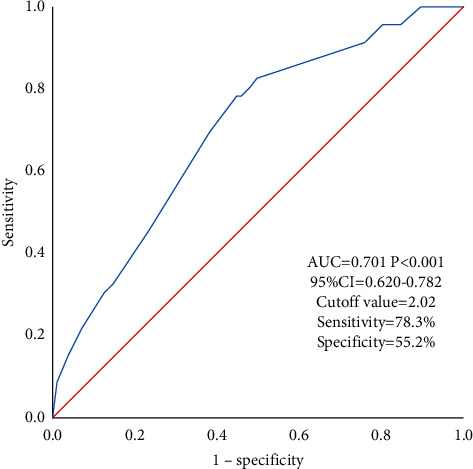
Receiver operating characteristic (ROC) curve to evaluate the combination prediction power of contralateral occult papillary thyroid cancer.

In univariate logistic regression analysis (Table 2), no significant association was found in diameter, LNM, and B-RAF mutation; hence, we excluded them from our multivariate logistic regression analysis. By multivariate analysis, variables significant in univariate analysis, including ipsilateral multifocality (OR=2.21, 95% CI=1.11–4.40, *P*=0.03), intact capsule (OR=2.54, 95% CI=1.24–5.19, *P*=0.01), CNM (OR=3.00, 95% CI=1.21–7.49, *P*=0.02), and HT (OR=2.08, 95% CI=.02–4.23, *P*=0.04), were also remarkably associated with COPTC. To visualize the prediction factors, we plotted a forest plot presented in Figure 2. Then based on the output of multivariate logistic regression, we had a ROC curve to evaluate the combining potency of the four statistically significant predicting factors and find an appropriate cutoff point, presented in Figure 3. In ROC analysis, the area under the curve (AUC) of the predicting model of contralateral occult carcinoma was 0.701(95% CI= 0.620–0.782, *P* < 0.001), and the Youden Index (0.335) was used to obtain the cutoff value of the model (cutoff = 2.02, sensitivity = 78.3%, and specificity = 55.2%), indicating the power of discriminating of the model is acceptable.

In Table 3, we further evaluated the association between the diameter of the occult lesions and the obtained variables but find that when 3 mm was used as the cutoff value, there was no statistical correlation between the diameter of occult tumor and the four obtained variables.

## 4. Discussion

Although the incidence of papillary thyroid cancer has increased worldwide over the recent years, the prognosis seems to remain stably excellent [19], and recurrence, rather than death, becomes the main burden of PTC patients, which partly has a relation with the approach and extent of initial surgery strategies. The option between total thyroidectomy and lobectomy plays a crucial role in discussing the strategy to treat papillary thyroid cancer. With increasing emphasis on risk-stratified management, lobectomy as an initial approach for unifocal tumors less than 4 cm without extrathyroidal extension (ETE) and LNM has been acceptable in some centers. With one lobe intact, patients may avoid complications relating to total thyroidectomy (such as hypocalcemia, hoarse, or even dysphonia) and prevent themselves from lifelong TSH repression therapy. Meanwhile, for those tumors < 1 cm (defined as papillary thyroid microcarcinoma, PTMC), milder tactics, always called active surveillance (AS), have been supported by some scholars for the reason of sluggishness of such mini tumors [20,21]. However, not all PTC has an excellent prognosis [22,23], and what is more important is that undetected contralateral occult thyroid carcinoma may lead to local-regional recurrence and even reoperation in those patients with clinical unilateral thyroid cancer. Furthermore, the precision of ultrasound in detecting lesions with diameters less than 5 mm was only 53.8% [10], and FNAC-diagnosed undetermined nodules also have a malignant risk of 18% or higher [24]. The effort to explore what kind of patients will benefit from total thyroidectomy is worthwhile.

In our study, the incidence of contralateral occult thyroid cancer is 20.1%. We found that multifocality of the ipsilateral lobe was an independent risk factor of the presence of COPTC in unilateral clinical PTC, meeting the same conclusion from some studies [25–28]. However, dissimilar to several previous studies [14,26], we excluded all the patients with contralateral nodules indicated by preoperative ultrasound, even those with TI-RADS < 3, for the reason of the limitation of the US to differentiate malignancies from benign nodules [29], which may lead to bias, and patients with occult contralateral benign nodules confirmed by postoperative pathology were assigned to control group. Several studies show that multifocality is an independent risk factor that predicts aggressive behavior and poor prognosis, even in terms of PTMC, for whom total thyroidectomy rather than lobectomy seems to be a preferable option. According to a large-scale cohort study from Kim [30], with a median follow-up period of 5 years, in patients who had a local recurrence, 58% of recurrent tumors were found in the contralateral lobes that had not been treated during the initial operation, and TT significantly reduced the risk of overall local, regional recurrence as well as outside of the remnant lobe in patients with multifocality (adjusted HR = 0.284, *P*=0.002; adjusted HR = 0.342, *P*=0.020, respectively). However, this conclusion was the opposite in a retrospective study by Harries [31], suggesting that multifocality did not increase overall recurrence. Until today, the role of multifocality in recurrence remains controversial, and further large-sample cohort studies are needed for more valuable evidence.

In this case-control study, we also found that the integrity of the tumor capsule and the metastasis of the central lymph nodes are both predictors of COPTC. Traditionally, tumor encapsulation was regarded as the boundary between benignity and malignancy [32]. In our practice, the capsular invasion was also seen as a symbol of intrathyroidal extension in PTC. Previous studies showed that encapsulation was correlated with multifocality and CNM [16,33,34]; therefore, we established a linear regression model based on these three variables to eliminate collinearity (VIF = 1.006 and 1.009, respectively). Furthermore, there were also studies [14,25,27], showing that capsular invasion was related to COPTC, which is contrary to our conclusion. That is interesting because some scholars defined multifocality as PTC with intrathyroidal extension with a hypothesis considering that the multiple foci result from a single primary lesion via intrathyroidal lymphatics [15]. However, there are also researchers who demonstrate that different foci may arise from different clonal copies that exhibit various biological behaviors from each other, underlying by some different gene mutations. In general, the causes of the multifocality of PTC are not clear. The volume may not present sequencing between the originally detected tumors and the COPTC. The value of capsular status needs to be discussed in the future.

Lymph node metastasis, especially CNM, where the most frequent recurrence occurs, is more common than an extrathyroidal extension or distant metastasis, the incidence of which reached up to 90%, and almost 50% of patients with PTC present with CNM when prophylactic central lymph node dissection (CND) is performed [35]. We have obtained that CNM is an independent risk factor for contralateral thyroid carcinoma, similar to recent studies [24,36]. However, LNM did not appear to show statistical significance between the case and control groups, probably because all patients who underwent bilateral lateral lymph node dissection were excluded because they met the excluding criteria, and ipsilateral lateral lymph node dissection can lead to the unknown status of the contralateral neck region.

A similar conclusion was obtained in terms of B-RAF mutation. B-RAF presents in 77.7% of all patients included in our study, which is a subtype of RAF kinase, as an activation of the MAPK pathway, which plays an essential role in regulating tumor growth and proliferation [37], indicating a higher risk of recurrence and a worse prognosis [5]. Kim et al. [17,38] suggested that the B-RAF mutation was an independent predictor of bilateral thyroid cancer. Similarly, in a study that included 248 patients [39], Yan et al. also suggested that the presence of the B-RAF mutation played a pivotal role in the prediction of bilateral thyroid cancer. However, in other studies, this relationship is not significant in accordance with another case-control study by Lee et al. [14], similar to our findings. This diversity may be due to our insufficient sample, and a larger sample scale may help us obtain a more accurate conclusion. In addition, tumor diameter has always received a lot of attention in the fields of thyroid cancer study. Usually, tumors were divided into PTMC and non-PTMC according to a diameter equal to 1 cm, indicating potential biological differences between these two subtypes of PTC. Previous studies showed that tumor > 1 cm was an independent factor of COPTC [29,36], but we did not reach the same conclusion. Further larger sample size multicenter studies may contribute to a powerful conclusion.

The extrathyroidal extension (ETE) has been recognized as an indicator of recurrence and death in differentiated thyroid cancer (DTC) [40]. The definition of ETE was the extension of the tumor outside the thyroid gland with invasion into surrounding structures such as the trachea, strap muscles, and recurrent laryngeal nerve (RLN). However, surgeons generally have difficulty differentiating between RLN and invasion of the trachea by the eyes. Consequently, RLN invasion was categorized as a subtype of trachea invasion. In our study, neither trachea nor strap muscle invasion was a significant indicator of the presence of COPTC. In fact, for patients with ETE, no matter if the association is absent or not, TT is always recommended, for ease of recurrence monitoring, and iodine therapy, due to the adverse future of such aggressive biological behaviors.

Hashimoto's thyroiditis is the most common autoimmune inflammatory disease of the thyroid, and the prevalence rate of PTC in patients with HT is 1.2% in fine-needle aspiration specimens and 27.6% in thyroidectomy specimens [41]. Some evidence has shown that autoimmune inflammation is a risk factor for thyroid cancer [42,43], while PTC patients with HT seem to have a better outcome [44]. Previous studies showed that compared to Graves' disease, patients with chronic lymphocytic thyroiditis are more likely to have incidental thyroid cancer [45]. In this study, we found that HT was an indicator of contralateral occult thyroid cancer. In fact, chronic autoimmune inflammation is widely recognized to cause precancerous conditions through the expression of inflammation genes and the induction of genomic instability. However, convincing mechanisms are still being debated, and whether autoimmune inflammation will lead to bilateral multifocal thyroid cancer is also an inevitable question that will require more biological research in the future. Furthermore, based on many experiences at our center, we found that the thyroid with HT always behaves in a blood-rich, bulky, and heterogeneous state, which can also lead to difficulties finding tiny lesions for sonographers.

There are some limitations to our study. Firstly, patients with PTC with solitary clinical lesions, no imaging evidence of lymph node metastases, and unfavorable behaviors were more prone to be performed lobectomy at our center, so those included in this study were all in advanced disease status, resulting in an overestimation of the predictive power of lymph node metastases. Secondly, considering advances in ultrasound technology and improved imaging diagnostics, only patients in the last two years were considered in our study, leading to a restricted sample size. Thirdly, active surveillance in the management of papillary thyroid microcarcinoma (always diameter < 1 cm) has been accepted by more and more scholars [46]. Since the occult lesions in the contralateral lobe were all less than 5 mm in this study, we used 3 mm as the cutoff value to conduct a correlation analysis between the discovered four independent risk factors and the diameter, but no statistical correlation was found. In fact, further prospective clinical studies are needed to determine whether such occult cancers need to be managed. Finally, because the risk factors we found depend on pathological determination, physicians' choice of final surgical method may rely on intraoperative frozen section, generating pressure on surgeons and patient concerns. Consequently, future researchers should explore imaging, serology, and gene-based prediction factors further.

## 5. Conclusions

PTC patients with multifocality in one lobe, intact capsule, central lymph node metastasis, or HT are more prone to harbor contralateral occult thyroid carcinoma. These patients should undergo more accurate clinical assessments to facilitate more appropriate surgical and follow-up procedures. Furthermore, further studies are also needed to find precise serum and genome indicators that predict COPTC.

## Figures and Tables

**Table 1:** Distribution of clinical and pathological characteristics in 229 patients.

Variables	Group		Statistical values		
	Case (N = 46)	Control (N = 183)	F	Chi-square	P
Age, years, mean ± SD	39.20 ± 11.65	39.06 ± 11.80	0.67		0.944

Gender, %				0.00	0.983
Female	32 (69.6)	127 (69.4)			
Male	14 (30.4)	56 (30.6)			

BMI, mean ± SD	24.00 ± 3.34	24.34 ± 3.94	1.47		0.592

Multifocality, %				5.59	<0.05
Presence	24 (52.2)	61 (33.3)			
Absence	22 (47.8)	122 (66.7)			

Location, %				0.28	0.597
Upper	12 (26.1)	55 (30.1)			
Otherwise	34 (73.9)	128 (69.9)			

Diameter, %				0.32	0.574
<1 cm	18 (39.1)	80 (43.7)			
≥1 cm	28 (60.9)	103 (56.3)			

Diameter of COPTC %					
<3 mm	36 (78.3)	—			
≥3 mm	10 (32.7)	—			

Capsule, %				5.64	<0.05
Intact	20 (43.5)	47 (25.7)			
Invasion	26 (56.5)	136 (74.3)			

Trachea invasion, %				0.14	0.706
Presence	3 (6.5)	15 (8.2)			
Absence	43 (93.5)	168 (91.8)			

Strap muscle invasion, %				0.10	0.753
Presence	5 (10.9)	23 (12.6)			
Absence	41 (89.1)	163 (87.4)			

CNM, %				4.63	<0.05
Presence	39 (84.8)	126 (68.9)			
Absence	7 (15.2)	57 (31.1)			

LNM, %				1.24	0.265
Presence	32 (69.6)	111 (60.7)			
Absence	14 (30.4)	72 (39.3)			

B-RAF mutation, %				0.13	0.716
Presence	35 (76.1)	143 (78.6)			
Absence	11 (23.9)	39 (21.4)			

Serum Tg^*∗*^, %				2.38	0.304
≤5 *μ*g/L	4 (10.3)	32 (20.4)			
5∼40 *μ*g/L	20 (51.2)	77 (49.0)			
>40 *μ*g/L	15 (38.4)	48 (30.6)			

HT, %				4.52	<0.05
Presence	20 (43.5)	50 (27.3)			
Absence	26 (56.5)	133 (72.7)			

^*∗*^Thirty-three patients did not have a preoperative serum thyroglobulin test.

**Table 2:** Univariate and multivariate analyses of predicting factors of COPTC.

Variables	Crude OR (95% CI)	P	Adjusted OR (95% CI)	B	Wald	P
Multifocality						
Absence	1		1			
Presence	2.18 (1.13–4.20)	0.02	2.21 (1.11–4.40)	0.79	5.06	0.03

Diameter						
< 1 cm	1					
≥ 1 cm	1.21 (0.62–2.34)	0.57				

Capsular						
Invasion	1		1			
Intact	2.23 (1.14–4.35)	0.02	2.54 (1.24–5.19)	0.93	6.61	0.01

CNM						
Absence	1		1			
Presence	2.52 (1.06–5.98)	0.04	3.00 (1.21–7.49)	1.10	5.57	0.02

LNM						
Absence	1	0.27				
Presence	1.48 (0.74–2.97)					

HT						
Absence	1		1			
Presence	2.05 (1.05–3.99)	0.04	2.08 (1.02–4.23)	0.73	4.09	0.04

B-RAF						
Negative	1	0.72				
Positive	0.87 (0.40–1.86)					

**Table 3:** Univariate and multivariate analyses between the diameter of the occult lesions and predictors.

Variables	Crude OR (95% CI)	P	Adjusted OR (95% CI)	P
Multifocality				
Absence	1		1	
Presence	1.50 (0.36–6.23)	0.58	1.46 (0.30–6.96)	0.64

Capsular				
Invasion	1		1	
Intact	2.09 (0.47–9.38)	0.34	2.19 (0.43–11.04)	0.34

CNM				
Absence	1		1	
Presence	0.65 (0.11–3.96)	0.64	0.33 (0.04–2.70)	0.30

HT				
Absence	1		1	
Presence	0.48 (0.11–2.15)	0.34	0.38 (0.07–1.99)	0.25

## Data Availability

The data that support the findings of this study are available from the corresponding author upon reasonable request.
